# International oncology drug approvals for multiregional or single-country clinical trials: A systematic review

**DOI:** 10.3389/fmed.2022.1084980

**Published:** 2022-12-15

**Authors:** Min Zhang, Igho Onakpoya, Katrin Rupalla

**Affiliations:** ^1^Nuffield Department of Primary Care Health Sciences, University of Oxford, Oxford, United Kingdom; ^2^Ymmunobio AG, Basel, Switzerland; ^3^Widler & Schiemann AG, Basel, Switzerland

**Keywords:** MRCTs, single-country, oncology drug approvals, time-to-approval, international

## Abstract

**Background:**

Cancer remains one of the most common causes of morbidity and mortality worldwide. Multiregional (MRCTs) and single-country clinical trials are two common approaches to support new oncology drug approvals internationally. However, systematic reviews comparing MRCTs with single-country trials for international oncology drug approval are lacking.

**Methods:**

We searched health agency websites to retrieve all approved oncology drugs from 2010 to 2022. ClinicalTrials.gov was used to retrieve all pivotal study information. We used an adapted version 2 of the Cochrane risk-of-bias tool for randomized trials (RoB 2) and Risk Of Bias In Non-randomized Studies - of Interventions (ROBINS-I) checklist to assess the risk-of-bias in randomized and non-randomized trials, respectively.

**Results:**

A total of 48 new drugs and biologics (comprising 215 pivotal clinical trials) with initial marketing approval in the United States, European Union, Japan, and China were included. The reporting quality of MRCTs vs. single-country studies was similar. The median time interval for approval was significantly longer for MRCTs than for single-country bridging studies (1,399 vs. 975 days, *P* < 0.0001), whereas the median time interval for approval was shorter for MRCTs than for single-country standalone studies. The time gap for oncology drugs approved before 2015 was significantly longer than for those approved after 2015. The median timeline for approval in MRCTs involving 3 regions showed the shortest time-to-approval compared with MRCTs involving 4–5 and 1–2 regions. There was no significant difference in the time-to-approval among different tumor types and product types.

**Conclusion:**

The median time-to-approval of MRCTs was significantly longer than that of single-country bridging studies but shorter than that of single-country standalone studies, primarily involving 3 regions as the most frequent pattern and the shortest time-to-approval to operate MRCTs as a pivotal trial. Single-country bridging studies still provide essential supplements for international oncology drug approvals if MRCTs do not apply. Future studies should explore how to shorten the time-to-approval for MRCTs.

**Systematic review registration:**

[https://www.researchregistry.com/browsethe-registry#registryofsystematicreviewsmeta-analyses/], identifier [1390].

## 1 Background

Cancer remains one of the most common causes of morbidity and mortality worldwide. In 2020, there were an estimated 18.1 million new cancer cases and 9.9 million cancer-related deaths (1). In 2040, the global cancer incidence is estimated as 30.2 and 16.3 million for mortality (2).

In 2020, top 10 cancer types for the estimated age-standardized mortality rates worldwide included lung (18%), colorectum (9.4%), liver (8.3%), stomach (7.7%), and breast (6.9%) (2). In the same year, Asia had the highest cancer mortality rate at 58.3%, followed by Europe (19.6%), Latin America and the Caribbean (7.2%), Africa (7.1%), and North America (7%). Lung cancer remains the leading cause of mortality in the United States (US), Europe, Japan, and China, whereas the other cancer types vary across these four regions (Table 1).

**TABLE 1:** Top 10 mortalities for cancers sites in the US, Europe, Japan, and China, both sexes, all ages in 2020.

Rank	US	EU	Japan	China
1	Lung (22.6%)	Lung (19.6%)	Lung (25.5%)	Lung (23.8%)
2	Colorectum (8.9%)	Colorectum (12.5%)	Colorectum (14.3%)	Liver (13%)
3	Pancreas (7.8%)	Breast (7.3%)	Stomach (11%)	Stomach (12.4%)
4	Breast (7%)	Pancreas (6.8%)	Pancreas (9.6%)	Esophagus (10%)
5	Prostate (5.3%)	Prostate (5.5%)	Liver (6.7%)	Colorectum (9.5%)
6	Liver (5.1%)	Stomach (5%)	Breast (4.1%)	Pancreas (4.1%)
7	Leukemia (3.9%)	Liver (4%)	Non-Hodgkin’s lymphoma (3.6%)	Breast (3.9%)
8	Non-Hodgkin lymphoma (3.4%)	Bladder (3.4%)	Prostate (3.2%)	Brain, central nervous system (2.2%)
9	Brain, central nervous system (3%)	Leukemia (3.2%)	Esophagus (2.9%)	Leukemia (2.1%)
10	Bladder (3%)	Kidney (2.8%)	Bladder (2.6%)	Cervix uteri (2%)

WCRF, (1) and GLOBOCAN, (21).

United States, China, Japan, and the European Union (EU) are the four largest pharmaceutical markets worldwide for market share (3). The world pharmaceutical market was estimated to be worthy of $1,077,856 million at ex-factory prices in 2020, among which, the North American market (the US and Canada) remained the world’s largest market with a 49.0% share, well ahead of Europe (23.9%), China (8.2%), and Japan (7.0%) (3). Therefore, the author has included these four regions as “international.”

The incidence of new cancer cases is estimated to increase by 59.2% in Asia, 21.0% in Europe, 37.9% in North America, 65.6% in Latin America and the Caribbean, and 89.1% in Africa by 2040, which indicates enormous burden on the global healthcare system. In addition, although the global numbers of treated patients have increased by an average of 4% annually from 2015 to 2020 (4), more cancer therapies to fulfill unmet needs (in most cases, 5-year survival < 30%) are in huge demand worldwide.

There remain substantial unmet needs for large cancer populations, including soft tissue sarcoma, gastrointestinal stromal tumors, triple-negative breast cancer, small cell lung cancer, ovarian cancer, uterine cancer, and esophageal cancer (3).

Cancer clinical trials are more complex in study design than other therapy areas, such as eligibility criteria, endpoints, and sites (4). Composite success rates in oncology have been treading down since 2015, with only 5.2% reported in 2021. The overall productivity of oncology research is among the lowest in the drug industry (4). Thus, clinical development strategy, including the selection of multiregional (MRCTs) or single-country clinical trials, is of great importance for global oncology drug development.

### 1.1 Approval of drugs in the US, EU, Japan, and China

An approved drug refers to a medicinal preparation validated for therapeutic use by a drug regulatory authority. New chemical entity (NCE) refers to a drug with no active moiety that the FDA has approved for other applications under section 505(b) of the Federal Food, Drug and Cosmetic Act (5). New molecule entity (NME) is a drug with an active moiety that has never been approved by the FDA or marketed in the US (6). NCE and NME are interchangeable, where NCE typically applies to chemical drugs, whereas NME applies to both chemical drugs and biologics. The US drug approval process takes place within a structured framework that includes analysis of the target condition and available treatments, assessment of benefits and risks from clinical data, and strategies for managing risks. In some cases, accelerated approval can be applied to promising therapies that treat a serious or life-threatening condition and provide therapeutic benefits over available therapies (7).

The US, EU, Japan, and China each have their own accelerated drug approval pathway (Table 2).

**TABLE 2:** Summary of drug accelerated approval pathway in the US, EU, Japan, and China.

Country/region	Accelerated approval pathway	Designation criteria	Year of implementation
US	Accelerated approval	1. Serious condition 2. Meaningful advantage over available therapy 3. Demonstrates an effect on an endpoint that is likely to predict clinical benefit	1992
EU	Conditional marketing authorization	1. Positive benefit-risk balance of the medicine 2. The applicant shall provide comprehensive data post-approval 3. The drug fulfills an unmet medical need	2004
Japan	Sakigake (22)	1. Innovative medical products 2. For serious diseases 3. Development and new drug application in Japan: being the world’s first or simultaneous with other countries 4. Prominent effectiveness expected in non-clinical and early phase clinical studies	2015
Japan	Conditional early approval (23)	1. Confirmatory clinical trials are not feasible. 2. Additional pharmacovigilance (PV) and risk minimization are required as approval conditions	2017
China	Conditional approval (24)	1. Drugs are indicated for treating severely life-threatening diseases without effective therapeutic options, for which clinical trials demonstrate efficacy and project clinical value. 2. Drugs urgently needed in public health, with efficacy evidence and clinical value projected by existing clinical trial data. 3. Vaccines urgently needed to respond to major public health emergencies or other vaccines considered urgently needed by National Health Commission with the benefits outweighing the risks	2017

### 1.2 Pivotal trials in the US, EU, Japan, and China

Drug review and approval are based on safety, efficacy, and quality through preclinical, clinical, and chemical, manufacturing, and controls studies. The US FDA defines pivotal clinical trials are human clinical trial intended for obtaining regulatory approval or confirming the safety and efficacy of the drug’s intended use (5). Japan’s defined a pivotal clinical trial as a critical clinical study for efficacy evaluation among submitted studies categorized as “major sources for evaluation” (8). The EU and China have no official definitions of pivotal clinical trials; however, the requirements for these are consistent with the specification in the USA and Japan (Table 3). Although the FDA traditionally required two pivotal trials for drug approval request, this requirement is to some extent diminished (9), especially in situations where drug sponsors have conducted multiple pivotal trials in their drug development programs (10).

**TABLE 3:** Definition of a pivotal clinical study.

List of countries or regions	Definition of pivotal clinical study
US	- A human clinical trial intended to be a pivotal trial for obtaining regulatory approval or - Any other clinical trial to confirm the safety and efficacy of the drug’s intended use.
EU	- No official definition for pivotal trials; however, they are consistent with the definition in US and Japan.
Japan	- A critical clinical study for efficacy evaluation among submitted studies categorized as “major sources for evaluation” (“HYOUKA SIRYO” in Japanese), as described in the PMDA review reports. If a clinical study was mentioned as a “reference” (“SANKO SIRYO” in Japanese) in a review report, the study was not classified as a pivotal clinical study (8).
China	- No official definition for pivotal trials; however, they are consistent with the definition in US and Japan

Pivotal clinical trials may be at any stage—Phase 1 (Ph1), 2, or 3—given the level of evidence as agreed by health agencies to support the drug’s marketing authorization. For any initial marketing approval, there might be single- or multiple pivotal clinical trials as evidence.

### 1.3 MRCTs

Multiregional are clinical trials conducted in more than one region under a single protocol that allow data from one country or region to help gain approval in another country or region (11). Multiple regions start the clinical trial, usually simultaneously, identified as the first subject first visit. A harmonized clinical study protocol is generally applied for MRCT with the same study design, such as eligibility criteria for participants, interventions, comparators, and endpoints. The number of enrolled participants is decided by the allocation plan in the statistical analysis plan. MRCTs usually consist of single-region MRCTs [i.e., China, Australia, and Republic of Korea as Asia-Pacific (APAC)–based MRCTs] or international MRCTs (i.e., US, EU, APAC, or other international regions). This review includes both single-region and international MRCTs.

A region refers to a geographical region, country, or regulatory region. A regulatory region comprises countries with a standard set of regulatory requirements for drug approval (e.g., the EU) (11). In this review, all EU-based clinical trials conducted in more than one EU country are counted as MRCTs, whereas clinical trials conducted only in the US, Japan, or China are not counted as MRCTs.

### 1.4 Single-country clinical trials

This refers to clinical trials conducted in a single country, whereas multiple centers may apply for a single-country clinical trial. A single-country clinical trial may be a bridging study for any other pivotal clinical trials or a standalone confirmatory trial conducted in any single country or region. The decision of whether an MRCT or a single-country clinical trial shall apply is based on different factors, which is discussed in the following sections.

### 1.5 Types of global clinical development strategies

There are typically four types of global clinical development strategies: simultaneous and synchronization for MRCTs and bridging and standalone for single-country studies (8).

#### 1.5.1 Simultaneous

Multiple countries or regions join the same clinical development program at the same time from an early stage until pivotal MRCT.

#### 1.5.2 Synchronization

Any country or region that has not joined early stage MRCT but caught up with pivotal MRCT later.

#### 1.5.3 Bridging

An additional study performed in a new region to provide pharmacokinetics (PK), pharmacodynamics, or clinical data on the efficacy, safety, dosage, and dose regimen of an innovative drug in the new region, which will allow extrapolation of foreign clinical data to the population in the new region (12). A bridging study can be initiated before the global first approval (pre-approval) or after any global first approval (post-approval) as two different approaches.

#### 1.5.4 Standalone

Any single-country clinical trial conducted independently from foreign clinical data. The clinical data obtained from the standalone clinical trial will typically support single-country drug approval only.

### 1.6 Why it is essential to do this review

There are several reasons why this systematic review has become imperative, which are mentioned below:

1. Composite success rates in oncology have been treading down since 2015, with only 5.2% in 2021. As a result, the overall productivity of oncology research is among the lowest in the industry (4). This review shall provide helpful insight to plan upcoming pivotal trials to support global oncology drug approval.

2. Clinical trial cost comprises 30–40% of the overall drug research and development cost (13), and the decision to conduct an MRCT or a single-country trial will broadly impact the overall cost of the drug development programs.

3. The decisions to conduct MRCTs or single-country trials are often irreversible; therefore, a right-first-time approach is expected for the clinical development plan.

4. The final drug approval by health agencies largely depends on sufficient evidence of drug safety, efficacy, and quality from either an MRCT or a single-country pivotal clinical trial.

Systematic reviews comparing MRCTs with single-country trials for international oncology drug approvals are lacking. This systematic review is the first one to study the time to global oncology drug approval under MRCTs or single-country studies as pivotal trials.

## 2 Methods

### 2.1 Search strategy

Electronic searches were conducted in the following databases for records from 1 January 2010 to 1 July 2022:

– FDA website for approved drugs for oncology (cancer)/hematologic malignancies since 2006.
– US FDA website.
– EU European Medicines Agency (EMA) website.
– Japan Pharmaceuticals and Medical Devices Agency (PMDA) List of Approved Products.
– Japan PMDA Review Reports (Drugs).
– China National Medical Products Administration (NMPA) website.
– China NMPA Center for Drug Evaluation.
– Clinical registry platform website.

Keywords such as oncology, cancer, hematology, hematologic malignancies, pivotal, pivotal study (studies), pivotal trial(s), and confirmatory were used to retrieve pivotal clinical studies from approved oncology drugs.

There were no language restrictions for the search results. The author has kept initial language search results from Japanese and Chinese websites.

### 2.2 Inclusion and exclusion criteria

This review only includes initial marketing authorization approval with the first indication approved in the above countries; it does not include any new indication or line extension applications as subsequent filings. A detailed list of eligibility criteria is presented below.

#### 2.2.1 Inclusion criteria

1. Drugs used for treating solid tumors or hematological malignancies that have obtained initial approval in the US, EU, Japan, and mainland China from 1 January 2010 to 1 July 2022 (cutoff date for data search and extraction).

2. Pivotal studies that have been initiated for approved oncology drugs in the US, EU, Japan, and China.

#### 2.2.2 Exclusion criteria

1. All generics for chemical drugs, biosimilars for therapeutic biologics, and medical devices.
2. Solid tumor or hematologic malignancy drugs that have not been approved in the EU, Japan, or mainland China.
3. Approved drugs that have been withdrawn from either the US, EU, Japan, or China.
4. Approved drugs as line extension (not initial application) from its first-approved non-oncology indication.
5. Studies not confirmed as pivotal trials for approved oncology drugs in the US, EU, Japan, or China.
6. Pivotal studies supporting indications that have been withdrawn from the US, EU, Japan, or China after initial approval.
7. Pivotal studies that have not yet started.
8. Pivotal studies initiated after drug approval as a post-marketing requirement or post-approval commitment studies.

### 2.3 Outcomes

#### 2.3.1 Primary outcomes

Time interval from the pivotal trial initiation to final drug approval in each country or region.

#### 2.3.2 Secondary outcomes

Time gap between the last drug approval date in one country and the earliest drug approval date in another country within the US, EU, Japan, or China.

### 2.4 Quality assessment

Since this review did not involve any individual patient data listings, one reviewer (MZ) used partial parameters (i.e., random sequence generation, allocation concealment, blinding of participants and personnel, and blinding of outcome assessment) from RoB 2 (14) to assess the risk-of-bias in randomized trials included in this review. For non-randomized trials, the author has used partial parameters (i.e., bias due to confounding, bias due to selection of participants, bias in the classification of interventions, bias due to deviations from intended interventions) from ROBINS-I (14) to assess the risk-of-bias in non-randomized trials included in this review. The author has also extracted the first literature reference from each disclosed study in ClinicalTrials.gov to conduct a quality assessment. A second reviewer (IO) independently verified the risk-of-bias and cross-checked the extracted data. Disagreements were resolved through discussion.

### 2.5 Data extraction

For each included pivotal trial, the following information was extracted:

1. Trade name (only US trade name included): Extracted from US approval notifications as a unique identifier. Trade names from other marketed countries were not extracted because they were not directly relevant to the outcome.
2. Company name [marketing authorization holder (MAH)]: Extracted from US approval notification. Specific MAH company name was not collected for products marketed in other countries because they were irrelevant to the study outcome.
3. INN (identifier for the approved product): Extracted from US approval notification as a unique identifier, which is identical throughout other countries as a unique identifier for the marketed drug.
4. Product type (chemical drug, biologics): Drug classification was extracted from agency review reports. This assumed the same drug classification applied throughout the US, EU, Japan, and mainland China.
5. Date of initial approval (dd-mm-yy), not counting any supplemental application approvals: Extracted from the agency review report.
6. Approval type (regular, accelerated, conditional), obtained from agency review report: Extract from agency review report.
7. Approved indication (consistent with approval label): Extracted from agency review report for the approved indication. For multiple indications, information was also extracted from corresponding countries.
8. Pivotal trial number [National Clinical Trial (NCT) number]: Used as a unique identifier for any pivotal study retrieved for every approval.
9. Pivotal trial start date (dd-mm-yy): Extracted from the “actual study start date” reported in the corresponding NCT# record.
10. List of countries in the pivotal trial: Retrieved from the “Locations” field within records from ClinicalTrials.gov. The number of geographical regions, countries, or regulatory regions was counted.
11. Pivotal trial type (MRCT/single-country), dependent on the list of countries involved in the pivotal trial: For a list of countries in the pivotal trial more than one, this was counted as MRCT. Pivotal trials in a single country were counted as a single-country trial.
12. Pivotal trial stage (1–4): Retrieved from the “Phase” column from each NCT# record.
13. Tumor type (provide a complete list, convert from approved indication): Extracted from #7 approved indication.

### 2.6 Data analysis

Summary tables were used to present the information from pivotal trials. Descriptive statistics [means, standard deviations (SD), median, 25% quantile (Q1), 75% quantile (Q3), minimum, maximum] were used to compute effect estimates. Shapiro–Wilk method was used for the normality test, and the Levene method was used for the homogeneity of variance test. Subsequently, *t*-tests (t statistic) were used for two-group comparisons and analyses of variance (F statistic) for multigroup comparisons to evaluate differences between groups when the data were normally distributed and following assumptions regarding the homogeneity of variance; otherwise, non-parametric methods were used [Wilcoxon rank sum tests (Z statistic) for two-group comparisons and Kruskal–Wallis H tests for multigroup comparisons (H statistic)]. For multigroup comparisons with a *P*-value < 0.05, the *p*-values in multiple comparison tests were corrected using the Bonferroni method. *P* < 0.05 was considered statistically significant. All analyses were performed using the Statistical Package for the Social Sciences (SPSS) Version 21.0 (SPSS Inc., Chicago, USA).

Subgroup analyses were performed based on the following three variables:

1. Product type (chemical drugs, biologics).
2. Tumor type (solid tumors, hematologic malignancies).
3. Number of different regions under MRCTs.

Data analysis was performed by one reviewer (MZ).

## 3 Results

### 3.1 Search results

**FIGURE 1:**
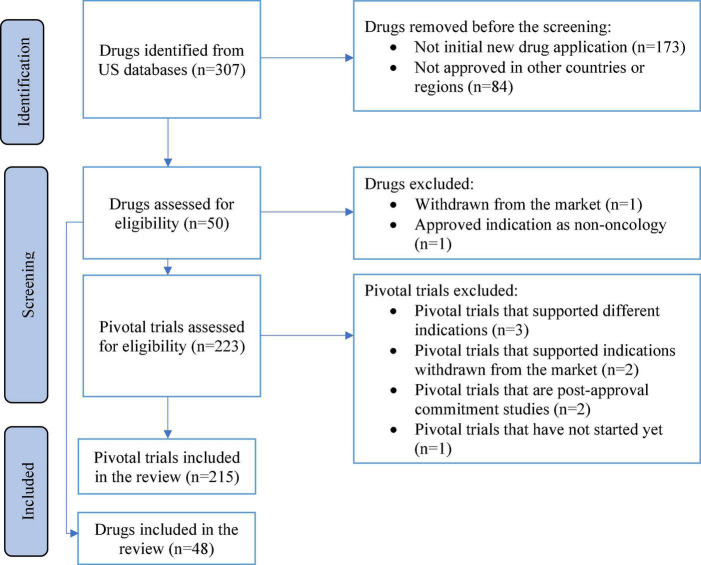
Flow diagram for the identification of studies.

The search procedure described in Figure 1 was applied. The search results are summarized below.

#### 3.1.1 Included studies

A total of 48 new drugs and biologics and 215 pivotal clinical trials were included.

#### 3.1.2 Excluded studies

A total of two approved drugs and eight pivotal trials are excluded.

### 3.2 Main characteristics of the included studies

A total of 48 new oncology drugs were approved in the US, EU, Japan, and China from 2010 to 2022, among which 67% (32/48) were chemical drugs and 33% (16/48) were biologics (Table 4). Regarding initially approved indications within these four regions, 56% (27/48) were for solid tumors and 44% (21/48) were for hematologic malignancies. Table 4 presents more details about the approved drugs.

**TABLE 4:** Characteristics of approved drugs.

Group	Number of approved drugs
**Drug types**	
Chemical drug	32 (67%)
Biologics	16 (33%)
**Indications**	
Solid tumors	27 (56%)
Hematologic malignancies	21 (44%)
**Approval types–regular approval**	
US	28 (58%)
EU	40 (83%)
Japan	47 (98%)
China	34 (71%)
**Approval types–accelerated approval/conditional approval**	
US	20 (42%)
EU	8 (17%)
Japan	1 (2%)
China	14 (29%)
Total	48

In the US, 42% (20/48) of the initial approvals were granted accelerated approval by the FDA, which aimed for earlier drug approval to treat severe conditions and fulfilled unmet medical needs based on surrogate endpoints (15).

In the EU, 17% (8/48) of the initial approval were granted conditional approval for life-threatening diseases, which include orphan medicines.

In Japan, only one conditional approval was granted among the total 48 approved oncology drugs: lorlatinib (by Pfizer) for treating ALK fusion gene–positive unresectable advanced and recurrent non-small cell lung cancer with resistance or intolerance to ALK tyrosine kinase inhibitor(s).

In mainland China, 29% (14/48) of the initial approvals were conditional, including two Hainan Special Access introduced after 2021 (16).

In total, 215 clinical trials were retrieved as pivotal clinical study data for the above 48 oncology drugs approved in the US, EU, Japan, and China.

Table 5 presents the characteristics of pivotal trials. Of these trials, 85% (183/215) were conducted as MRCTs and 15% (32/215) as single-country clinical trials. Among the MRCT pivotal trials, 32% (58/183) were Ph1, Ph2, or Ph1/2 stage and 68% (125/183) were Ph3 or Ph2/3 stage. Among the 32 single-country clinical trials, 88% (28/32) were Ph1, Ph2, or Ph1/2 stage and 12% (4/32) were Ph3 stage. In addition, only one was conducted in the US (NCT02631044, lisocabtagene maraleucel, DLBCL), 16 trials in Japan, and 11 in China. All these four single-country Ph3 pivotal studies were conducted in mainland China after the first global approval, which were NCT01695135 (Abiraterone Acetate Tablets, mCRPC), NCT03476239 (Blinatumomab for Injection, ALL), NCT03029234 (Carfilzomib for Injection, Multiple Myeloma), and NCT02225470 (Eribulin Mesilate Injection, Breast Cancer).

**TABLE 5:** Characteristics of pivotal trials.

Group	MRCT	Single
**By countries**		
China	37 (20%)	15 (47%)
EU	56 (31%)	0
Japan	38 (21%)	16 (50%)
US	52 (28%))	1 (3%)
**By product types**		
Biologics	60 (33%)	12 (38%)
Chemical drug	123 (67%)	20 (62%)
**By tumor types**		
Hematologic malignancies	51 (28%)	10 (31%)
Solid tumors	132 (72%)	22 (69%)
**By study phase**		
Phase 1–2	58 (32%)	28 (88%)
Phase 3	125 (68%)	4 (12%)
**By number of regions**		
1	15 (8%)	–
2	26 (14%)	–
3	71 (39%)	–
4	47 (26%)	–
5	24 (13%)	–
Total	183 (85%)	32 (15%)

Among the single-region MRCT pivotal trials, 67% (10/15) were conducted in mainland China and other APAC countries (such as Republic of Korea, Hong Kong, and Taiwan). For MRCT with two (geographic) regions, 65% (17/26) were conducted in the US or Canada and EU, 8% (2/26) in the APAC countries and US, and 27% (7/26) in the APAC countries and EU. MRCTs involving 3 (geographic) regions was the most frequent approach and comprised 39% (71/183) of all MRCTs. Among these, 94% (67/71) were conducted in the US, EU, and APAC countries; 5% (3/71) in the US, the EU, and South America; and 1% (1/71) in the US, the EU, and Africa.

For all MRCT pivotal trials, simultaneous development strategy was the most frequent approach, applied in 80% of the trials (146/183; Table 6).

**TABLE 6:** Summary of development strategy based on MRCTs/single-country studies.

Count of pivotal trial number	Approval country				
**Development strategy**	**China**	**EU**	**Japan**	**US**	**Total**
**MRCT**	**37**	**56**	**38**	**52**	**183**
Simultaneous	10	55	29	52	146 (80%)
Synchronization	27	1	9	0	37 (20%)
**Single**	**15**	**0**	**16**	**1**	**32**
Bridging	11	0	16	0	27 (84%)
Standalone	4	0	0	1	5 (16%)

For single-country pivotal trials conducted in China only, 73% (11/15) were bridging studies with PK elements that aimed to achieve extrapolation from global approval, among which 36% (4/11) followed the pre-approval approach (prior to any global first approval) and 64% (7/11) followed the post-approval approach from global first approval. Only four pivotal trials were under the Ph3 stage as standalone development to verify safety and efficacy among the Chinese population, which were indicated for breast cancer, metastatic castration-resistant prostate cancer, multiple myeloma, and acute lymphoblastic leukemia.

### 3.3 Risk-of-bias

**FIGURE 2:**
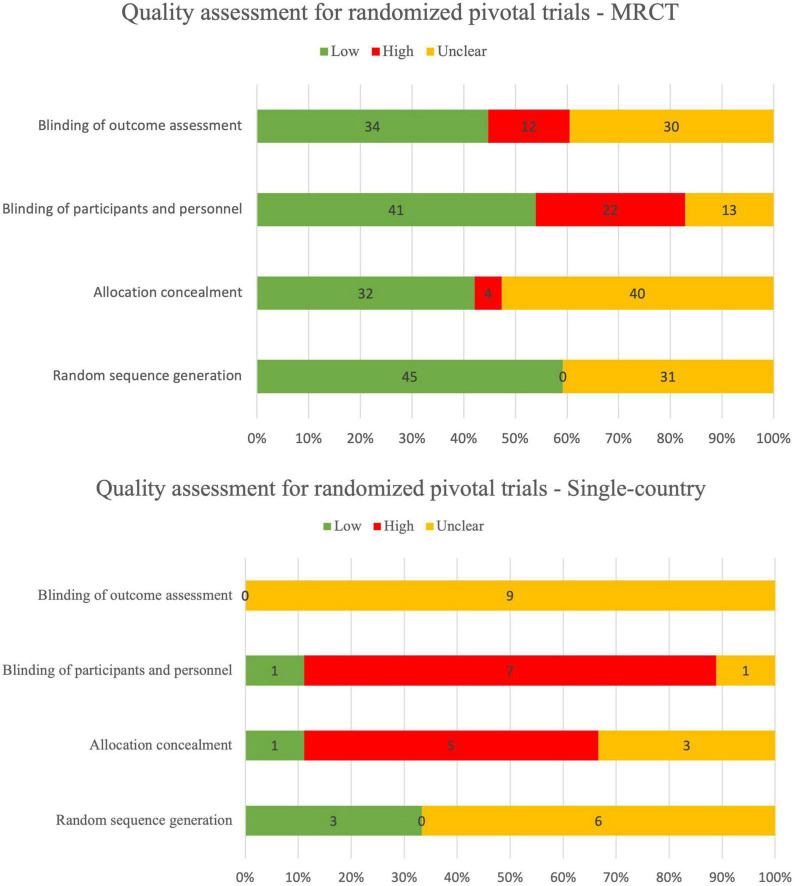
Quality assessment for randomized pivotal trials using RoB 2.

**FIGURE 3:**
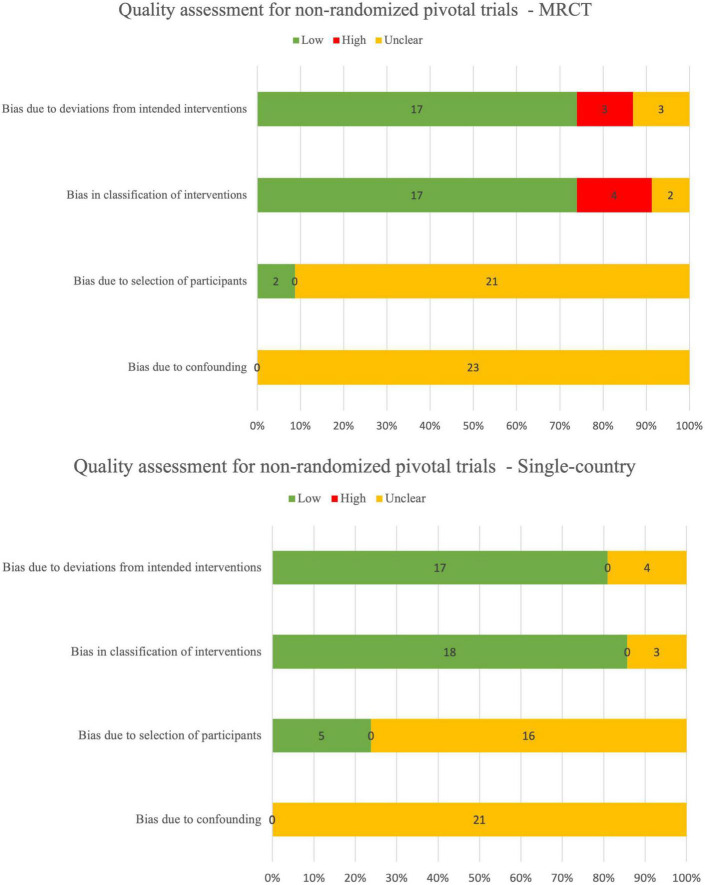
Quality assessment for non-randomized pivotal trials using ROBINS-I.

There were 27/80 (34%) randomized pivotal trials that were identified as low risk-of-bias, 11/80 (14%) as low-to-moderate, 17/80 (21%) as moderate, 15/80 (19%) as moderate-to-high, 7/80 (9%) as high risk, and 3/80 (4%) as unclear risk-of-bias (Figure 2). There were 3/49 (6%) non-randomized pivotal trials identified as low risk-of-bias, 29/49 (59%) as low-to-medium, 6/49 (12%) as moderate, 7/49 (14%) as moderate-to-high, and 4/49 (8%) as high risk-of-bias (Figure 3).

Among the 80 randomized pivotal trials, 75/80 (94%) were MRCTs and 5/85 (6%) were single-country trials. Among the 49 non-randomized pivotal trials, 24/49 (49%) were MRCTs and 25/49 (51%) were single-country trials.

Overall, the reporting quality of between MRCT and single-country was similar, both for randomized controlled trials (RCTs) and non-RCTs. Quality assessment range distribution for MRCTs and single-country trials is summarized in Table 7.

**TABLE 7:** Summary table for risk-of-bias for MRCTs and single-country trials.

Study type	High	Low	Low-to-moderate	Moderate	Moderate-to-high	Unclear	Grand total
MRCT	9 (9%)	27 (27%)	26 (26%)	17 (17%)	18 (18%)	2 (2%)	99
Single-country	2 (7%)	3 (10%)	14 (47%)	6 (20%)	4 (13%)	1 (3%)	30

### 3.4 Outcomes reporting

#### 3.4.1 Time interval for approved oncology drugs between MRCTs and single-country trials

The median time interval for approval in MRCTs was 1,399 days (Q1, Q3: 1135.00, 1782.00), which was significantly longer than that in single-country (bridging) trials (1,399 vs. 975, Bonferroni corrected *P* < 0.0001). However, the difference in the timeline for approval was not statistically significant between MRCTs and single-country (standalone) (Bonferroni corrected *P* = 1.000) and single-country (bridging) and single-country (standalone) (Bonferroni corrected *P* = 0.065) trials (Table 8).

**TABLE 8:** Time interval for approved oncology drugs between MRCTs and single-country trials.

Time interval for approval	MRCT	Single-country (bridging)	Single-country (standalone)	Statistic	*P*-value
N (missing)	183 (0)	27 (0)	5 (0)	*H* = 21.50	<0.0001
Mean (SD)	1472.98 (530.45)	1022.22 (330.00)	1529.20 (465.41)		
Median	1399.00	975.00	1558.00		
Q1, Q3	1135.00, 1782.00	758.00, 1207.00	1141.00, 1832.00		
Min, Max	291, 3618	579.00, 1785.00	1000.00, 2115.00		

#### 3.4.2 Time gaps for approved oncology drugs between MRCT and single-country trials

We chose 2015 as the cutoff year to explore the trend of global oncology drug approval gaps. The rationale is mainly due to the regulatory reform for drug and medical devices in mainland China since 2015. The time gap of approval in the US/EU/Japan/China for oncology drugs approved before 2015 was significantly longer than that for drugs approved after 2015. The median gap times in trials approved before 2015 and after 2015 were 2,032 and 1,037 days, respectively (Table 9).

**TABLE 9:** Time gaps for approved oncology drugs before 2015 and after 2015.

Time gaps for approval	Before 2015	After 2015	Statistic	*P*-value
N (missing)	25 (0)	23 (0)	*Z* = 5.06	<0.0001
Mean (SD)	2147.24 (732.80)	985.52 (475.46)		
Median	2032.00	1037.00		
Q1, Q3	1469.00, 2635.00	553.00, 1331.00		
Min, Max	1188.00, 3728.00	230.00, 1792.00		

### 3.5 Subgroup analysis

No significant difference was noted in the timeline of approval among different tumor types (*P* = 0.0840) or drug types (*P* = 0.4658) (Tables 10, 11).

**TABLE 10:** Time interval for approval between different tumor types.

Time interval for approval	Hematologic malignancies	Solid tumors	Statistic	*P*-value
N (missing)	62 (0)	153 (0)	*Z* = 1.73	0.0840
Mean (SD)	1359.81 (566.96)	1441.13 (512.04)		
Median	1255.50	1420.00		
Q1, Q3	1019.00, 1465.00	1089.00, 1759.00		
Min, Max	434.00, 3618.00	291.00, 2763.00		

**TABLE 11:** Time interval for approval between different drug types.

Time interval for approval	Biologics	Chemical drug	Statistic	*P*-value
N (missing)	72 (0)	143 (0)	*Z* = 0.73	0.4658
Mean (SD)	1409.44 (603.31)	1421.83 (488.62)		
Median	1321.5	1395		
Q1, Q3	993.00, 1663.50	1089.00, 1702.00		
Min, Max	374.00, 3618.00	291.00, 2763.00		

In multiple comparison test, the median timeline for approval for MRCTs involving 4–5 regions was significantly longer than that for MRCTs involving 3 regions (1,482 vs. 1,337; Bonferroni corrected *P* = 0.049). No statistically significant difference was noted between MRCTs involving 1–2 regions and those involving 3 regions (Bonferroni corrected *P* = 1.000) or between MRCTs involving 1–2 regions and those involving 4–5 regions (Bonferroni corrected *P* = 0.188) (Table 12).

**TABLE 12:** Time interval for approval in MRCTs with different numbers of regions.

Time interval for approval	1–2 regions	3 regions	4–5 regions	Statistic	*P*-value
N (missing)	41 (0)	71 (0)	71 (0)	*H* = 6.32	0.0425
Mean (SD)	1421.80 (497.59)	1401.55 (523.56)	1573.96 (546.73)		
Median	1358	1337	1482		
Q1, Q3	1024.00, 1816.00	1092.00, 1632.00	1243.00, 1841.00		
Min, Max	639.00, 2500.00	374.00, 3618.00	291.00, 3427.00		

## 4 Discussion

### 4.1 Summary of main findings

The main objective of this review was to explore the patterns of approvals across geographical regions for MRCTs vs. single-country trials. The reporting quality of MRCTs vs. single-country trials was similar. The longer time interval for oncology drugs with MRCTs than with single-country bridging studies was mainly because (1) 69% (22/32) of the single-country trials were initiated after global first approval for oncology drugs and (2) 88% (28/32) of the single-country trials were bridging studies with only PK elements, which were less complex than MRCT pivotal trials with both efficacy and safety elements.

The lack of statistical difference between MRCTs and single-country (standalone) trials and between single-country (bridging) and single-country (standalone) trials may be due to low statistical power because of the small number of available single-country standalone studies. Nevertheless, the median time interval for approval in MRCTs was still shorter than that in single-country standalone studies, which demonstrates the value of MRCTs in accelerating global oncology drug approvals compared with single-country standalone studies. Moreover, clinical evidence gained from MRCTs shall support more regions than single-country standalone studies, which typically will support its country drug approval only.

No significant difference was noted in the timeline of approval among different tumor or drug types, indicating that a similar approach to drug development strategy can apply to various types of oncology indications and different modalities of drugs. However, the sponsor shall consider those novel modalities, such as cell therapy products, in terms of their global development strategy and how MRCT shall apply. The only single-country standalone trial for the US was from a cell therapy treatment (lisocabtagene maraleucel), which may be driven by the logistics challenge for MRCT conduction for such kind of products (17).

The time gap of approval in the US/EU/Japan/China for oncology drugs approved before 2015 was significantly longer than that for drugs approved after 2015. This was mainly driven by a regulatory reform in China in 2015 when the local health agency allowed China’s participation in global development programs before the first global approval of the drug.

The median timeline for approval in MRCTs involving 3 regions was significantly shorter than that for MRCTs involving 4–5 regions and shorter than that for MRCTs involving 1–2 regions, which suggests that MRCTs involving 3 regions might be the most efficient approach. The author assumes that the involvement of more regions within MRCTs shall bring more complexity and take more time than MRCTs involving fewer regions. The shorter median timeline for approval in MRCTs involving 3 regions than those involving 1–2 regions indicates that more variety of ethnic groups of patient enrollments may accelerate global oncology drug approvals. Among the MRCTs involving 3 regions, 94% (67/71) were conducted in the US, EU and APAC countries, covering the top 3 estimated numbers of new oncology cases.

The combination of the US, EU, and APAC populations also reflects the global epidemiology profile for high cancer incidence and mortality rates in the APAC, EU, and US regions.

The US has the highest number of accelerated approvals (42%), which is because of its earlier launch of drug accelerated development programs since 1992. On the other hand, the higher percentages of conditional approvals granted in China (29%) compared with the EU (17%) and Japan (2%) are mainly attributed to the drug and medical device regulatory reform since 2015 to shorten significant drug lags for globally available treatments that are not accessible in China (18), as well as the launch of “List of Urgent clinical needs drugs that are approved outside of China” since 2018, which encouraged companies to register globally approved drugs in China with potential local clinical trial waiver (conditional approval) to fulfill unmet medical needs. As a result, 96% (12/14) of these conditional approvals were granted after 2018 after the implementation of this list. However, overall, mainland China still lags behind the US, EU, and Japan for new oncology drug approvals; 94% (45/48) of new oncology drugs approval in China are later than those in the US, the EU, and Japan.

The US, the EU, and Japan have joined the same pivotal study development program more frequently since they are all listed as the International Council for Harmonisation of Technical Requirements for Pharmaceuticals for Human Use (ICH) founding members with harmonized drug development standards. China followed through the synchronization approach by either joining an additional APAC-based MRCT involving 1–2 regions or other international MRCTs involving 3–5 regions. The relatively lower number of simultaneous (10/37) and a more significant number of synchronizations MRCTs (27/37) conducted in China were mainly due to the historic lengthy clinical trial approval duration before 2018, which took around 12–15 months to get Clinical Trial Application (CTA) clearance before China can join any MRCT (18). Cases for China’s removal from the initial list of MRCT involved countries including NCT01801111, Alectinib Hydrochloride Capsules, Ph1 and 2 MRCT, initiated from 20 June 2013, and NCT01010061, Obinutuzumab Injection, Ph3 MRCT, initiated from 21 December 2009.

### 4.2 Comparison with the existing literature

Two-thirds of oncology novel active substance launches have been for solid tumors in recent years, with 68 launches in the last 5 years, up from 35 in the 5 years prior (4). Regarding initially approved indications within these four regions, 71% (34/48) were for solid tumors and 29% (14/48) were for hematologic malignancies. The result is consistent with the overall proportion of solid tumors approved globally by the IQVIA report.

Mckinsey’s report highlighted that the increased competition had shortened development cycles, and competitor therapies rapidly followed an initial launch. Table 9 shows that global oncology drug approval lags as few as less than 1 year were initially approved on 27 September 2018 (dacomitinib tablets) and 28 May 2021 (sotorasib), whereas the most significant approval gap of > 2,000 days was initially approved before 2015. This was mainly due to China’s drug and medical device reform since 2015 and the official rollout of the ICH E17 MRCT guideline worldwide after 2017. Therefore, the author cannot conclude whether the increased competition has shortened development cycles. Furthermore, this review does not include any lifecycle management applications, generics or biosimilar products, and their relevant pivotal trials.

Our review result is consistent with the trend of earlier trials from McKinsey’s report (3), which highlighted that success in this environment requires exploring new development paradigms, including placing a greater emphasis on earlier trials (as 54% of approvals for assets with a breakthrough designation are based on Ph1 or 2 studies), site-agnostic approvals (such as Keytruda for MSI-H or dMMR mutations and Vitrakvi for NTRK mutations), and exploration of real-world evidence for expanding indications for a particular treatment (for example, prescribing Ibrance for male breast cancer or ongoing Opdivo ATTRACTION-2 study). From this review, the higher numbers of Ph1 and 2 trials approved in the US (*n* = 22) and EU (*n* = 21) than in China (*n* = 6) and Japan (*n* = 9) suggest that the US and EU have been more proactively seeking earlier clinical development for marketing approval, with the FDA/EMA seeming more supportive in accepting earlier phase of a clinical trial as a pivotal trial for oncology drug approval. The acceptance of earlier phases of pivotal trials will typically lead to accelerated approval in the US or conditional approval in the EU to fulfill unmet cancer treatment needs. On the other hand, the fewer number of earlier phases of a pivotal trial in Japan and China is mainly due to the later implementation of the conditional approval pathway in Japan (2017) and China (2020).

We also compared this review with two other reviews (19, 20). The first article referred to real-world evidence to support the US original and supplemental approval of oncology from 2015 to 2020 and concluded that external control real-world studies complemented efficacy data from single-arm trials for US oncology product approvals (19). The second article aimed to characterize pivotal trials supporting supplemental new indication approvals of drugs by the US FDA from 2017 to 2019 and concluded that there was little difference in the design characteristics of the pivotal trials supporting supplemental indication and original approvals (20). These two articles retrieved a more comprehensive range of oncology drug approvals in the US for both original and supplemental approvals. However, the search period was shorter than this review. Therefore, the conclusions of these two reviews are somewhat consistent with the findings of this review. However, those two reviews did not retrieve any approval timelines.

### 4.3 Strengths and limitations

This systematic review is the first to study the time-to-approval for global oncology drugs under MRCTs or single-country studies as pivotal trials. The search covered a wide range of new oncology drugs throughout the four largest geographic regions as representatives for global pharmaceutical markets in the last decade. The retrieved drugs and pivotal studies provided the most relevant evidence to reflect the current drug modalities (chemical drugs and biologics) and assessment of pivotal clinical trials as the pillar for international oncology drug approvals by health agencies. In addition, the reporting quality of the included studies was assessed.

However, the limitations of this review include the selection of four representative countries and regions that may not reflect the whole international drug approval landscape. As a result, some potentially relevant studies may have been missed during searches. The exclusion of pivotal trials that support subsequent approvals such as additional indications may also limit the extent to which such pivotal studies impact MRCTs and single-country clinical trials for oncology drug approval. The double counting for the same MRCTs for different country marketing approval may also cause confusion, although it does not impact result part and is necessary for data analysis for this review. Moreover, the impact of the COVID-19 pandemic worldwide and uncertainties in geographic conflicts (i.e., Russia, Ukraine, etc.) are not assessed in the current review.

### 4.4 Implications for research

Multiregional is the most frequent pattern for pivotal trials to support global oncology drug approvals, regardless of chemical drugs or biologics and solid tumors or hematologic malignancies, based on the search results of this review. With the full implementation of ICH E17 from 2017, it is estimated that pharmaceutical companies shall keep up the trend of applying MRCTs for oncology drugs that aim for global approval. Although the time-to-approval is significantly longer for MRCTs than for single-country bridging studies, the significant drug gaps between first-country approval and the target country are still the major disadvantage of the single-country bridging strategy. Therefore, drug companies may consider single-country bridging studies as a supplementary strategy if target countries cannot join MRCT pivotal trials. There have been only five single-country standalone pivotal trials for global oncology approval since 2010, showing that this development strategy is not typical for global oncology drug approval. The single-country standalone strategy is usually considered if a bridging strategy is not applicable or if a standalone confirmatory trial is required by a health agency for the target country.

Our review also shows that MRCTs involving 3 regions are potentially the most efficient approach for pivotal trials that support global oncology drug approvals. Time-to-approval has not shown a significant difference between MRCTs involving 3 regions and MRCTs involving 1–2 regions. However, MRCTs involving 3 regions shall include more diverse ethnic patient groups that support drug approvals for more countries, considering that the US, EU, and APAC regions comprise a majority of MRCTs involving 3 regions. Drug companies shall consider how to streamline clinical operations for MRCTs to shorten the time-to-approval for future global oncology development.

Although the quality assessment shows similarities between MRCTs and single-country trials, the overall reporting quality of disclosed pivotal trials needs to be improved by sponsors and applicants for both MRCTs and single-country trials, regardless of randomized or non-randomized approach.

From the health agency perspective, they have been accepting foreign clinical data started with adopting ICH E5 standards as the basis for a single-country bridging study to fill the gap between first approval countries and target countries. In addition, implementing ICH E17 standards from 2017 will further promote MRCTs as the best practice for global pivotal clinical trials in oncology. Meanwhile, the collaboration among international regulators may also allow patients with cancer to receive earlier drug access in other countries with the potential delay of drug approvals. For example, the US FDA has introduced Project Orbis through which pharmaceutical companies were asked to submit applications for clinically significant oncology drugs to participating countries simultaneously for concurrent review by their regulatory authorities. The US FDA has also implemented the Real-Time Oncology Review initiative to provide a more efficient review process to ensure earlier access to safe and effective patient treatment.

Although the research is only focusing on global oncology drug approvals, the authors assume that the findings shall also apply to non-oncology drug approvals and pivotal clinical trials in general regardless of disease types and product types. The management of trials, namely, clinical trial operation may be the key factor for time to approval variances across different MRCTs. The selection of investigational sites, patient screening and recruitment procedure, site trainings and monitoring are some of the potential holding items that need further investigation in future research.

The optimal design of pivotal trials may also be another key holding item. It may be driven by the establishment of more excellent uniformity of new global standards of treatment and a deeper understanding of variations in medical practice across different countries and regions.

## 5 Conclusion

The median time-to-approval for MRCTs is significantly longer than for single-country bridging studies and shorter than for single-country standalone studies. In addition, there is no difference in the time-to-approval for different product types or tumor types, which indicates that the MRCT development strategy shall apply to all drug modalities for different tumor indications.

The reporting quality is similar for MRCTs vs. single-country trials. MRCT involving 3 regions is the most frequent pattern to conduct MRCTs as pivotal trials, and it also demonstrates the shortest time-to-approval compared with MRCTs involving 4–5 and 1–2 regions. The US, EU, and APAC are the most frequent combination of MRCTs involving 3 regions. Single-country clinical trials as bridging studies still provide essential supplements if MRCTs do not apply. Single-country bridging studies and acceptance of foreign clinical data are recommended to shorten drug lags and potentially shorten drug approval time.

Despite no significant difference between MRCTs and single-country standalone studies for time-to-approval due to the limited number of single-country standalone trials, MRCTs still show a shorter median time-to-approval than single-country standalone studies. Meanwhile, MRCTs show an advantage in supporting drug approval in multiple regions simultaneously, whereas single-country standalone studies can only support drug approval for their own countries in most cases. There is a trend of an earlier phase of MRCT pivotal trial (i.e., Ph1, 2) to support oncology drug approval internationally, especially in the US and EU. However, the accelerated or conditional approval based on Ph1 or 2 studies are still subject to confirmatory trial for conversion into regular approvals. The reporting quality of future pivotal trials should be improved. Future research should focus on how to accelerate MRCT pivotal trials to support global oncology drugs.

## Data availability statement

The original contributions presented in this study are included in the article/supplementary material, further inquiries can be directed to the corresponding author.

## Author contributions

MZ: protocol development, database searches, data extraction and quality assessment, data-analysis and interpretation, and drafting of the review. IO: protocol development, quality assessment, data-analysis and interpretation, and co-drafting of the review. KR: protocol development, initial database searches, data extraction, and data interpretation. All authors contributed to the article and approved the submitted version.
